# Intelligence Beliefs Predict Spatial Performance in Virtual Environments and Graphical Creativity Performance

**DOI:** 10.3389/fpsyg.2021.671635

**Published:** 2021-08-23

**Authors:** Jon-Chao Hong, Jian-Hong Ye, Mei-Lien Chen, Jhen-Ni Ye, Ling-Wen Kung

**Affiliations:** ^1^Institute for Research Excellence in Learning Sciences, National Taiwan Normal University, Taipei, Taiwan; ^2^Faculty of Education, Beijing Normal University, Beijing, China; ^3^Dhurakij Pundit University, Bangkok, Thailand; ^4^Department of Industrial Education, National Taiwan Normal University, Taipei, Taiwan; ^5^Graduate Institute of Technological and Vocational Education, National Taipei University of Technology, Taipei, Taiwan

**Keywords:** graphical creative performance, implicit theories of intelligence, spatial performance, virtual reality, intelligence belief

## Abstract

Although intelligence beliefs have been applied to explain the influence of cognition, behavior, and creativity, the research on creativity is still limited. Therefore, in order to effectively expand the understanding of the influence of intelligence beliefs on the creative performance of learners’ graphics, the implicit theories of intelligence were exploited as the basis of this study. Three hypothetical pathways were proposed to be explored, and a research model was validated. First- and second-year students from a technical high school in New Taipei City were invited to participate. There were 273 valid data (88.9% of complete data). Reliability and validity analyses were performed, as well as overall model fit analysis and research model validation, and descriptive statistical analysis of the learners’ performance in applying the operational virtual reality (VR). The results of this study showed that: (1) Incremental beliefs of aesthetic intelligence had a positive effect on spatial performance; (2) entity belief of spatial intelligence (EBSI) had a negative effect on spatial performance; and (3) spatial performance had a positive effect on graphical design performance. From the results, it is clear that design teachers can assess students’ implicit beliefs in the early stages of teaching to actively promote better spatial performance when students show high levels of entity beliefs.

## Introduction

Social psychology aims to illustrate how human beliefs profoundly influence behavior. Beliefs are critical to learners’ enthusiasm for learning and their ability to maintain perseverance and growth in the face of challenges and difficulties (Dweck, 2002). Therefore, Dweck (2000) proposed a social cognitive approach to the study of human intelligence through implicit theories which emphasize that humans can define intelligence using incremental or entity belief from implicit theories of intelligence (ITI). For example, proponents of incremental beliefs consider that human intelligence attributes are malleable and can be improved through effort; conversely, proponents of entity beliefs consider that human intelligence attributes are fixed and cannot be easily changed (Murphy and Dweck, 2016; Schroder et al., 2019). Various theories of intelligence beliefs can produce predictive effects on cognition, emotion, and behavior in different contexts (Spinath et al., 2003). Therefore, for decades, implicit theories have been supported by many studies, confirming that the theories can continue to be developed in a variety of fields (Huang et al., 2017). Accordingly, this study analyzes the relevant creative performance based on Dweck (2000, 2006) ITI.

Previous studies have demonstrated a close relationship between executive functions and thought performance. For example, there are two types of executive experiences when performing thinking tasks, such as reasoning, problem solving, and decision making. Type 1 processes information in an intuitive and associative manner without capacity constraints, whereas Type 2 involves reflexive, analytical, and logical processes, and its executive processes depend on cognitive resources (Lin et al., 2018), which echoes the perceived degree of two types of intelligent implicit beliefs. Specifically, executive function is a collective term for a series of constituent processes, such as planning, adjustment, working memory, and constraint, that are necessary to successfully regulate thought and goal-oriented intelligent behavior (Scibinetti et al., 2011). Executive functions (EFs) are thus the basic cognitive processes controlling thought and action (Benedek et al., 2014). Therefore, the present study is based on the theory of incremental intelligence, which was constructed to investigate the effects of spatial play and attentional functioning on creative performance during executive function.

The studies suggest that the theory of intelligence can be used in realistic settings and has a positive impact on achievement performance (Blackwell et al., 2007), while it also points out that intelligence and creativity are interrelated constructs (Benedek et al., 2014), so using implicit intelligence beliefs as the theoretical basis for creativity research will help strengthen the research discourse. Creativity is a complex and multifaceted phenomenon and an executive function that can cognitively relate phenomena to various ways of expressing new concepts (Sharma and Babu, 2017). Therefore, researchers can study creativity from various perspectives, including how personal beliefs affect creativity (Intasao and Hao, 2018), as one study suggests that an important part of understanding creativity is through self-reflection on the cognitive components of spatial and aesthetic thinking.^1^ In addition, a previous study has shown the benefits of digital technology on students’ creative development (Bereczki and Karpati, 2021). Therefore, a virtual reality (VR) device for assessing participants’ spatial performance ability (SPA) was applied in this study to explore how spatial ability can impact creativity in graphic design. Accordingly, this study constructed a hypothetical model to examine the relationship between beliefs of aesthetic and spatial intelligence, spatial performance, and graphical creativity performance (GCP) of current technical high school students through model validation.

## Research Method

### Research Hypotheses and Model

#### The Relationship Between Incremental Belief of Aesthetic Intelligence and Spatial Performance

In the argument of the ITI, the belief of incremental intelligence is that intelligence is malleable and can be expanded through acquired learning and experience, so this belief helps learners to achieve successful learning and good academic achievement (Rattan et al., 2015). In addition, studies have also shown that learners who hold beliefs about the development of intelligence are more likely to invest more effort and focus on learning goals to enhance their intelligence because they believe that intelligence is malleable (Kray and Haselhuhn, 2007; Rai and Lin, 2019). Furthermore, learners who hold beliefs about incremental intelligence will increase their abilities through continuous effort because they believe that abilities can be increased through acquired effort (Lüftenegger et al., 2019).

Previous research suggests that if learners tend to support beliefs about incremental intelligence, these beliefs motivate learners to face challenges, and learners tend to set learning goals that improve their abilities over time and work to increase their abilities and find solutions when they encounter difficulties (Mangels et al., 2006; Liu et al., 2014). Moreover, learners’ beliefs about their abilities will significantly affect their motivation to learn (Rieche et al., 2019). Therefore, learners who hold beliefs about incremental intelligence have better academic achievement on challenging campuses and have better completion rates in school (Blackwell et al., 2007; Yeager and Dweck, 2012).

Because learners with a belief in incremental intelligence view intelligence as malleable, they will strive to improve their intelligence in specific domains, and this belief is thought to be effective in terms of predicting learner achievement (Braasch et al., 2014). Furthermore, previous research has also confirmed that beliefs about incremental intelligence (positive beliefs) have a positive effect on learning outcomes (Lou et al., 2017). Taken together, when participants are learners with incremental belief, their achievement performance increases. In this study, aesthetic intelligence refers to the ability to perceive aesthetics from the psyche and to evaluate people and things aesthetically, while incremental belief of aesthetic intelligence (IBAI) is defined as the ability to perceive beauty. It is malleable and can be helped to grow through later experiences. Based on this, this study proposes the following research hypotheses on the relationship between IBAI and SPA.

*H1*: IBAI is positively related to SPA.

#### The Relationship Between EBSI and SPA

Learners who have entity beliefs consider that intelligence is not malleable and does not increase over time, and does not grow through effort and learning, and therefore, they are not committed to learning to improve their intelligence (Bruning et al., 2011; Haimovitz et al., 2011; Zonnefeld, 2019). Moreover, according to Dweck et al. (2008), although the ITI can positively influence learners’ learning behaviors, it can also have negative effects, especially when learners perceive themselves as having entity beliefs and are concerned about the complexity of the products they use and lack motivation to learn (Sharifi and Palmeira, 2017). Additionally, it has also been shown that learners who hold the entity belief of intelligence have lower learning performance when faced with failure (Dweck and Leggett, 1988).

Dweck (1986, 2000, 2012) noted that proponents of entity belief of intelligence believe that they can learn new things, including skills or knowledge. However, spatial ability may not be directly related to spatial intelligence beliefs, and the degree of spatial intelligence never changes (Furnham, 2014). Apart from this, it has also been shown that while students’ beliefs and goals can have a strong influence on their learning success, those students with a belief that intelligence is fixed are more likely to emphasize “performance goals,” causing them to be vulnerable to negative feedback and possibly choosing to avoid challenging learning opportunities as a result (Mangels et al., 2006) and performance goals are the goals that learners have for themselves in a particular subject, so that entity belief of intelligence is often associated with negative learning experiences or performance. Therefore, research suggests that entity belief of intelligence is effective in terms of predicting negative learning outcomes (Lou et al., 2017). In summary, when people believe that intelligence is innate and cannot be easily changed through learning, their achievement performance is likely to be poorer. Spatial intelligence in this study refers to abilities involving visual and spatial recognition, and graphical processing, whereas the EBSI is the belief that spatial intelligence is innate and cannot be refined through acquired learning or experience. Accordingly, this study proposed the following hypothesis for the study of EBSI on SPA:

*H2*: EBSI is negatively related to SPA.

#### The Relationship Between SPA and GCP

Spatial abilities can be referred to as the cognitive process of locating targets in space, perceiving distance and directional relationships, and mentally transforming their location (Lawton, 2010). Spatial ability represents an individual’s ability to visualize, maintain, and manipulate mental images in space (Yılmaz, 2017). Moreover, spatial ability has a significant impact on the performance of individuals, and on solidified or virtual objects in thinking about discrimination (Höffler and Leutner, 2011). In the field of education, spatial ability refers to the processing ability that helps learners to think spatially (Cheng, 2017). While measuring spatial ability, tasks are used to assess the spatial skills of the subjects, such as wayfinding or navigation in a virtual environment or outside the laboratory (Lawton and Kallai, 2002). In this study, spatial ability refers to the ability to process information involving space, and spatial performance refers to the performance of the participant when performing the spatial ability in the VR assessment system. In this study, SPA was measured using the results of VR spatial ability.

Creativity is often illustrated using terms, such as innovation, originality, and intelligence, where the role of creativity is represented in terms of these concepts (Runco, 2014). Previous studies have investigated domain-specific *vs*. domain-general creativity from a variety of perspectives. Researchers who use self-report measures of creativity are more likely to find evidence of domain generality. Domain-specific creativity is often loosely defined as large cognitive areas, such as mathematics and science (e.g., Huang et al., 2017; Sun et al., 2020), whereas performance-based creativity usually acquires evidence for assessing domain-specific creativity by a few narrowly defined activities, such as music (e.g., Podlipniak, 2021).

Students have been found to be interested in the possibility of integrating art and design into STEM courses as a way to enhance creativity (Perignat and Katz-Buonincontro, 2019). When integrating arts into an informal STEM module, Thuneberg et al. (2018) found that variables of relative autonomy, visual reasoning, and formal operations were related to creativity. Moreover, in their study of the acquisition of domain knowledge with divergent thinking for scientific creativity, Sun et al. (2020) found that students with higher levels of domain knowledge benefitted more than students with lower levels. Similarly, one study highlighted that metacognitive knowledge can boost students’ creativity in language learning because the students are effective in using strategies due to their awareness and control of their cognitive resources.

There are many existing domain-general creativity assessment tests, including the Guildford Battery Creativity Test (Guilford, 1967), which measures five main constructs, namely, fluency, flexibility, novelty, elaboration, and sensitivity. Another is the Torrance Test of Creative Thinking (Torrance, 1966). It has been used to assess six constructs: fluency, flexibility, novelty, elaboration, inventiveness, and penetration. Kim (2006) raised the use of creativity tests and suggested that creativity values could be adjusted for specific situations. Moreover, design mainly emphasizes the novelty of the product and its integration into a domain-specific context or field (Doboli et al., 2015). Creativity is also considered as a key factor in the design advertising process (Klebba and Tierney, 1995).

Design activities are considered to be an activity process involving needs analysis and problem clarification, information gathering and research, conceptualization and creative thinking, information generation and analysis, evaluation, and optimization (Lang et al., 2002). An engineering design study suggested that the process of innovation involves processing a large amount of design-related information in order to produce creative design outcomes, so the creative design process was conceptualized as the result of a consensus based on the integration of innovative design processes with cognitive psychology (Howard et al., 2008). In this study, domain-specific creativity refers to the novelty results of the participants’ GCP.

Additionally, spatial ability is considered as a cognitive ability (Tarampi et al., 2016). Is spatial ability critical to spatial design creativity? Many researchers have investigated the potential relationship between spatial ability and design performance and have found inconsistencies (Suh and Cho, 2020). For example, previous research suggests that spatial ability plays a critical and unique role in constructing many important psychological phenomena, that it has a unique role to play in the development of creativity, that design creation is a creative process (Kell et al., 2013), and that creativity has been linked to visual perception through past research (Suh and Cho, 2020). To sum up, when participants have good SPA, they are more likely to produce high levels of domain-specific creativity in design activities that require high levels of cognitive processing. Therefore, to understand the correlates between SPA and GCP, the following hypothesis was proposed.

*H3*: SPA is positively related to GCP.

### Research Model

**Figure 1:**
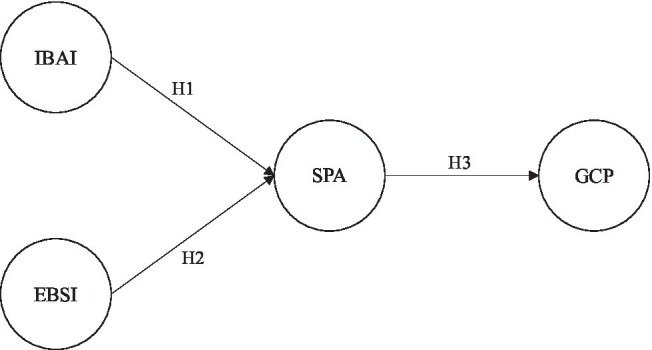
Research model. IBAI, incremental belief of aesthetic intelligence; EBSI, entity belief of spatial intelligence; SPA, spatial performance; and GCP, graphical creativity performance.

Hegarty (2010) identified two basic components of human intelligence: the first being mental imagery (e.g., aesthetic intelligence) and the second being more analytical forms of thinking (e.g., spatial intelligence). This review study points out that mental simulation is an important strategy in spatial thinking, which will be enhanced by more analytical strategies, for example, task decomposition and rule-based reasoning, including the ability to select the best external performance for a task and to use novel external representations effectively (Hegarty, 2010). In addition, a scaled research model must consider more than just a single intelligence (Bryan and Mayer, 2020), so both intelligences required for creativity in the creative domain of graphic design were considered in this study. In this study, the theoretical framework of the ITI proposed by Dweck (2000) was used to investigate the relationship between IBAI, EBSI, SPA, and GCP, as shown in Figure 1.

### Research Procedure

This study targeted participants from a technical high school in New Taipei City who were majoring in art and design. To recruit participants, first, we obtained the agreement of the school principal, school supervisors, and teachers to carry out the experiment. Second, students who were willing to join this study were asked to sign a consent form. Due to VR being a novel technology for these students, their willingness to take part in the study was very high, so no other incentives were needed to encourage participation.

**Figure 2:**
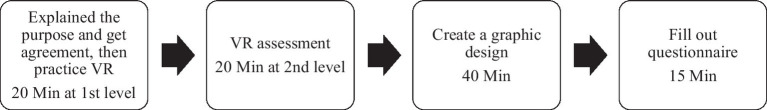
Procedure.

**Figure 3:**
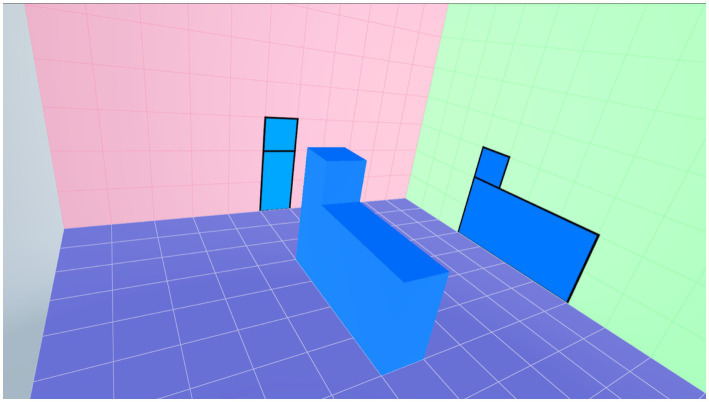
An exemplary screenshot of the first level of spatial assessment.

**Figure 4:**
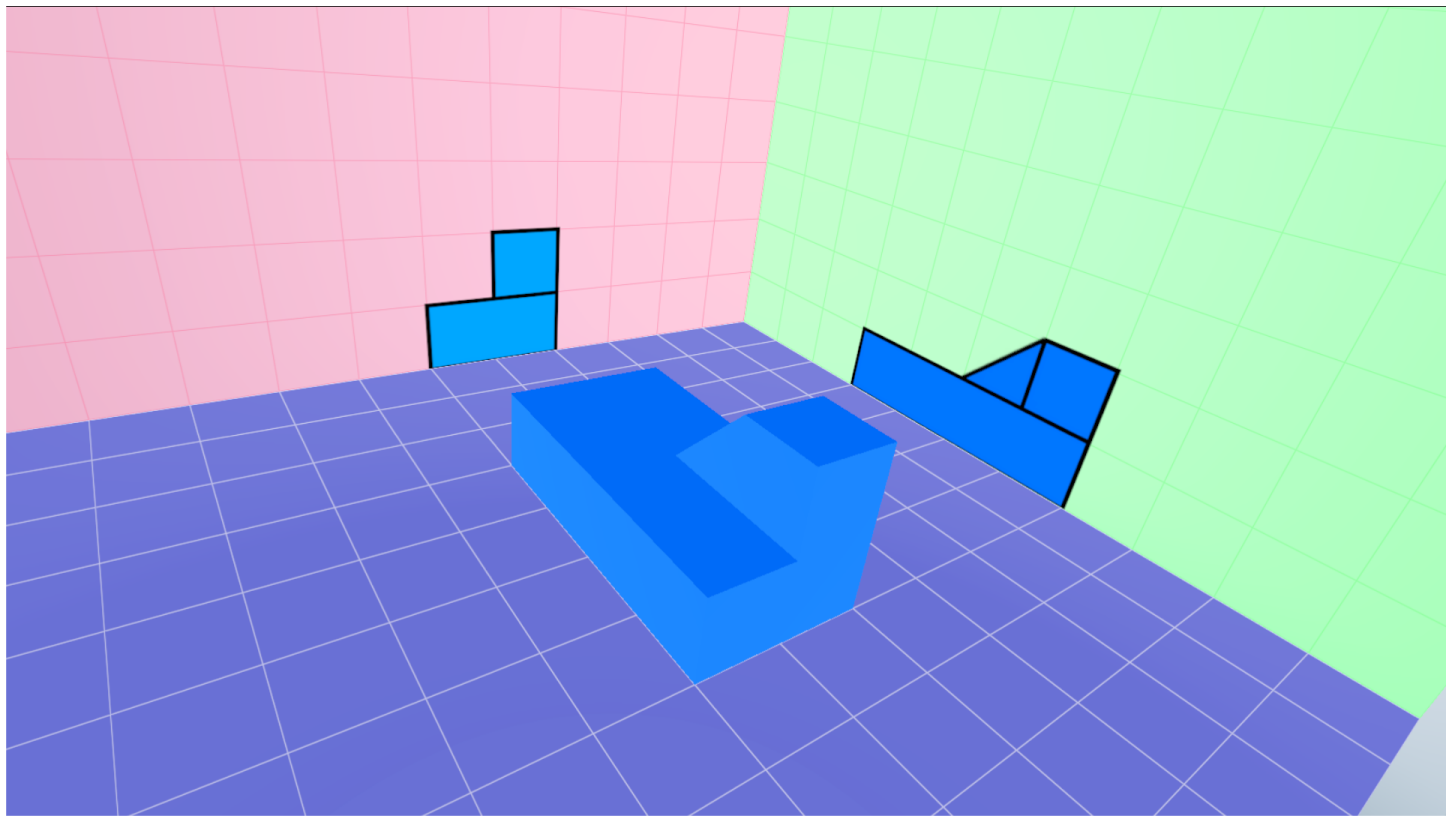
An exemplary screenshot of the second level of spatial assessment.

The implementation of the survey was divided into three parts, totaling 95min, as shown in Figure 2. First, the researcher explained the purpose of the study, introduced the computer hardware, and showed the VR operation video and the use of the “Spatial Assessment on VR” software and equipment to the participants. They were able to practice playing three sample stages at the first level to familiarize them with the operation and interaction mode of the VR spatial assessment, as shown in Figure 3. Second, each participant was given 20min to perform the VR spatial assessment. In this stage, participants started working from the first-level performance for assessing their beginning ability and then moved up to the second level, as shown in Figure 4.

**Figure 5:**
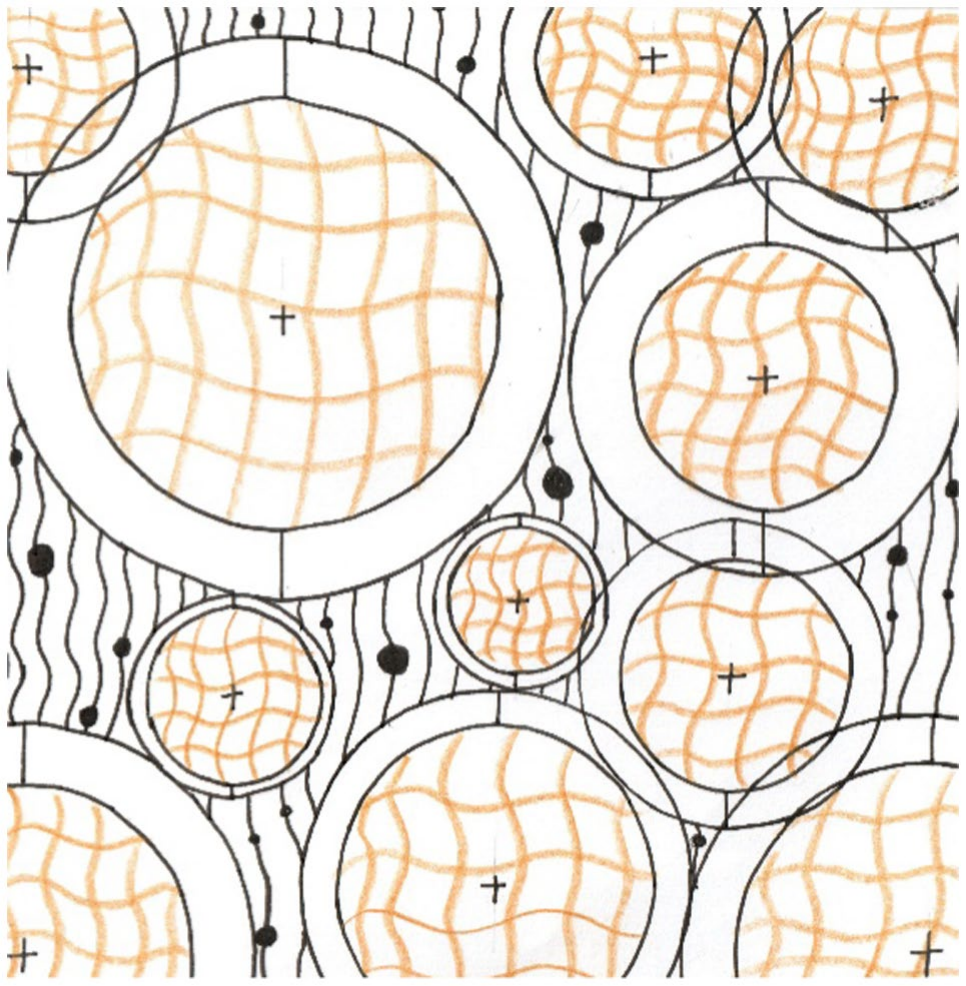
Graphical example.

After completing the VR spatial assessment, participants were given 40min to create a graphic design. At this stage, they were provided with design templates, such as a four-sided continuous graphic, a single graphic, and multiple graphics for reference, so that they could understand the type of graphic design. However, there was no restriction on the theme and scope of their creation in this study, as shown in Figure 5.

Finally, after the participants finished their design creations, the research questionnaire was distributed, and each participant was given 15min to complete it. Considering ethical requirements, participants were informed that they could leave the questionnaire blank if they did not want to answer it. Moreover, if they had any questions or did not understand any items in the questionnaire, they could directly ask the on-site study implementation staff, as shown in Figure 2.

### Participants

In this study, 308 sophomore and junior students of a technical high school in New Taipei City were invited to participate in this study by intentional sampling. Of the collected questionnaires, 35 were deleted due to incomplete responses or participants dropping out of the study. The total number of valid participants was therefore 273, with a percentage of complete data of 88.6%, including 56 male participants (20.5%) and 217 female participants (79.5%); 139 first-year participants (50.9%) and 134s-year participants (49.1%); and 51 participants with previous experience of VR (18.7%) and 222 without (81.3%). In addition, 144 participants had experience of playing 3D games (52.7%), and 129 participants did not (47.3%); while 154 participants usually played games (56.4%), and 119 participants did not (43.6%). The average age of the participants was 15.79years (standard deviation of. 78years).

### Measurement

The measurement for this study was translated and developed from previous studies and was reviewed by three educational psychologists for expert review to ensure the content validity of the instrument. The questionnaire was first given to students whose backgrounds were similar to the target samples to try out. It was revised accordingly to ensure face validity. The instrument was administered on a 5-point Likert scale, with 1 representing *strongly disagree*, 2 representing *disagree*, 3 representing *neutral*, 4 representing *agree*, and 5 representing *strongly agree*.

#### Incremental Belief of Aesthetic Intelligence

Students who hold implicit incremental beliefs consider that intelligence or specific abilities are a developable quality (van Aalderen-Smeets and van der Molen, 2018). In this study, aesthetic intelligence refers to the ability to perceive aesthetics from the psyche and to evaluate people and things aesthetically, while IBAI is defined as the ability to perceive beauty. It is malleable and can be helped to grow through later experiences. According to this definition, this study adapted from Dweck et al. (1995) Implicit Intelligence Scale, which measures participants’ perceptions of their beliefs about the development of aesthetic intelligence based on items, such as: Human aesthetic intelligence is changeable. This scale has six items. The internal reliability of the items ranged from. 0.94 to 0.98, the retest reliability was. 0.80, and the factor loadings were above. 0.90.

#### Entity Belief of Spatial Intelligence

Research has indicated that students with an entity belief in intelligence believe that intelligence cannot be acquired through effort and learning, nor can it be acquired over time (Haimovitz et al., 2011). Spatial intelligence in this study refers to abilities involving visual, spatial recognition, and graphical processing, whereas EBSI implies that spatial intelligence is innate and cannot be refined through acquired learning or experience. Therefore, based on this definition, the present study adapted Dweck et al. (1995) Implicit Intelligence Scale, with items, such as: “Human spatial intelligence is innate and I cannot do much to change it” to measure participants’ perceptions of spatial intelligence crystallization beliefs. This scale has six items. The internal reliability of the items ranged from 0.94 to 0.98, the retest reliability was 0.80, and the factor loadings were above 0.90.

#### Graphical Creativity Performance

Creative work is the result of an extended production process (Sawyer, 2018), and ideas are considered creative when they relate to useful artifacts while feeling new and surprising in the context of a particular social practice (Heinze, 2013). In this study, innovative creativity refers to the novelty results of the participants’ GCP. Therefore, this study used Hong et al.’s (2019) novelty measure of GCP to measure participants’ GCP. A 5-point scale was used, with (1) indicating “I have seen it before,” (2) indicating “I seem to have seen it,” (3) indicating “I’m not sure if I have seen it,” (4) indicating “I do not remember seeing it,” and (5) indicating “I have never seen it before.” One of the most popular ways to assess creativity is to rely on judgment, often asking professionals or other experts to judge creative works (Runco et al., 1994). Therefore, in this study, the GCP scores were evaluated by two professors in the design field and one designer with more than 10years of design work experience.

Prior to the formal evaluation, a 2-h meeting was held to explain the purpose of the study. Then, 10 templates were provided for the three raters to familiarize themselves with the scoring criteria. When there were inconsistencies in the scoring, a discussion was held so that a consensus could be reached. In order to achieve independence of scoring, the experts were given their own scores during the formal scoring and after all the scoring was completed. Spearman’s rank correlation coefficient was first applied for intra-rater reliability to confirm the consistency between the scores. Then, the scores of the three raters were summed and averaged to obtain the GCP of the participants.

#### Spatial Performance Ability

It has become a common feature in many games and platform systems to record players’ game data. Players can now track their achievements and analyze their past game behaviors (Medler, 2011). The SPA used in this study was calculated from the web backend of the SPA assessment VR system and the results of players’ game play, with a minimum cumulative score of 0, representing “did not pass any stage,” and a maximum score of 21, representing “passed all stages in this level.” The participants’ SPA scores were calculated based on the evaluation results within 20min.

**Figure 6:**
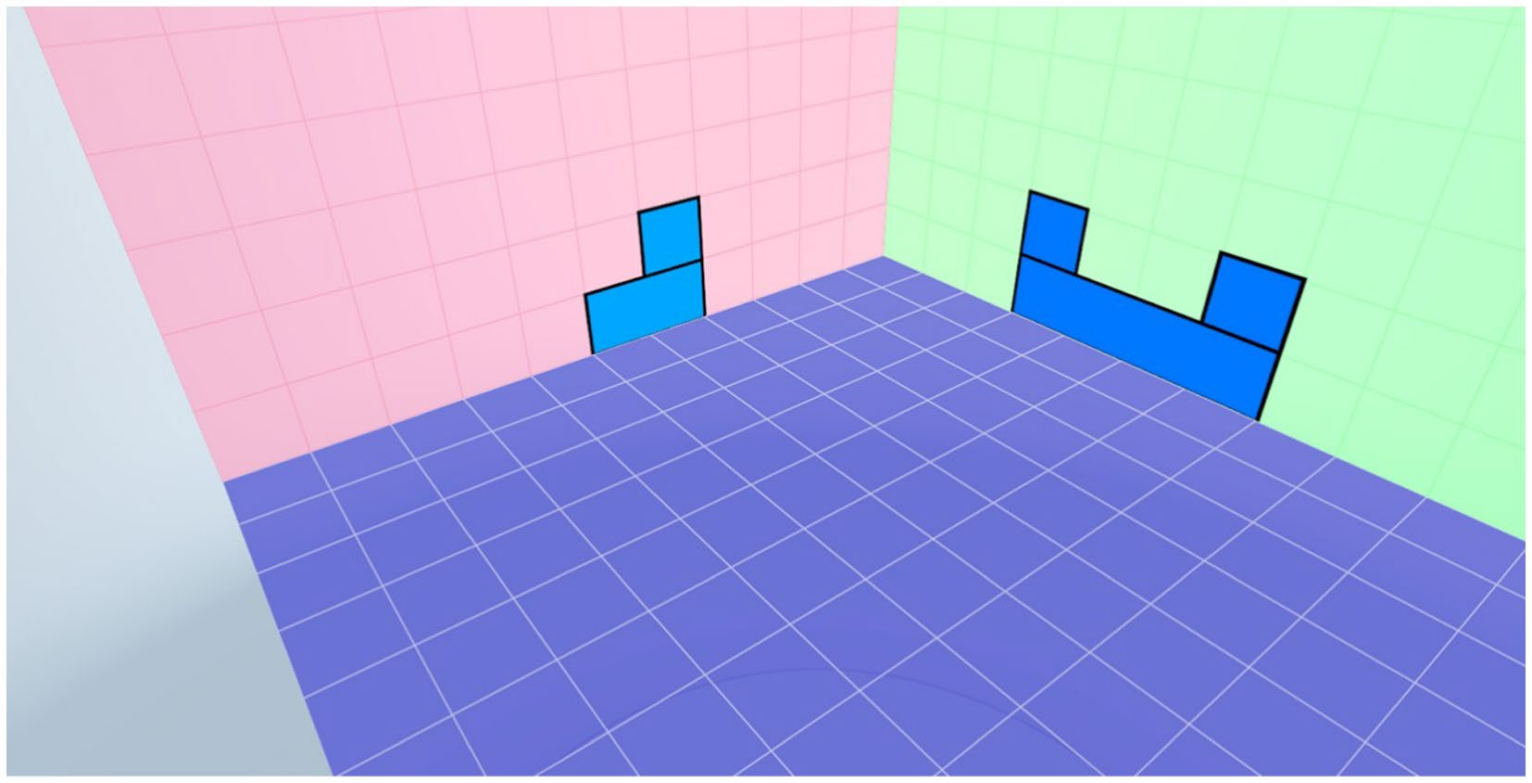
A screenshot of target dimensions: VR assessment example.

In the spatial performance assessment VR system, users need to be able to accurately perceive spatial properties, such as shape, size, plane, and spatial location of a visual image (spatial perception), to rotate spatial shapes in their minds (mental rotation), and to accurately perceive visual space and translate what they know into solutions (spatial visualization) (Linn and Petersen, 1985). In other words, in the test task, VR provided the user with a front view, a side view, and a set of blocks, and the user had to arrange the corresponding three-dimensional shapes according to the front and side views, meaning that the three-dimensional blocks had to correspond exactly to the projected positions and should not be placed under the horizontal plane, as shown in Figure 6.

**Figure 7:**
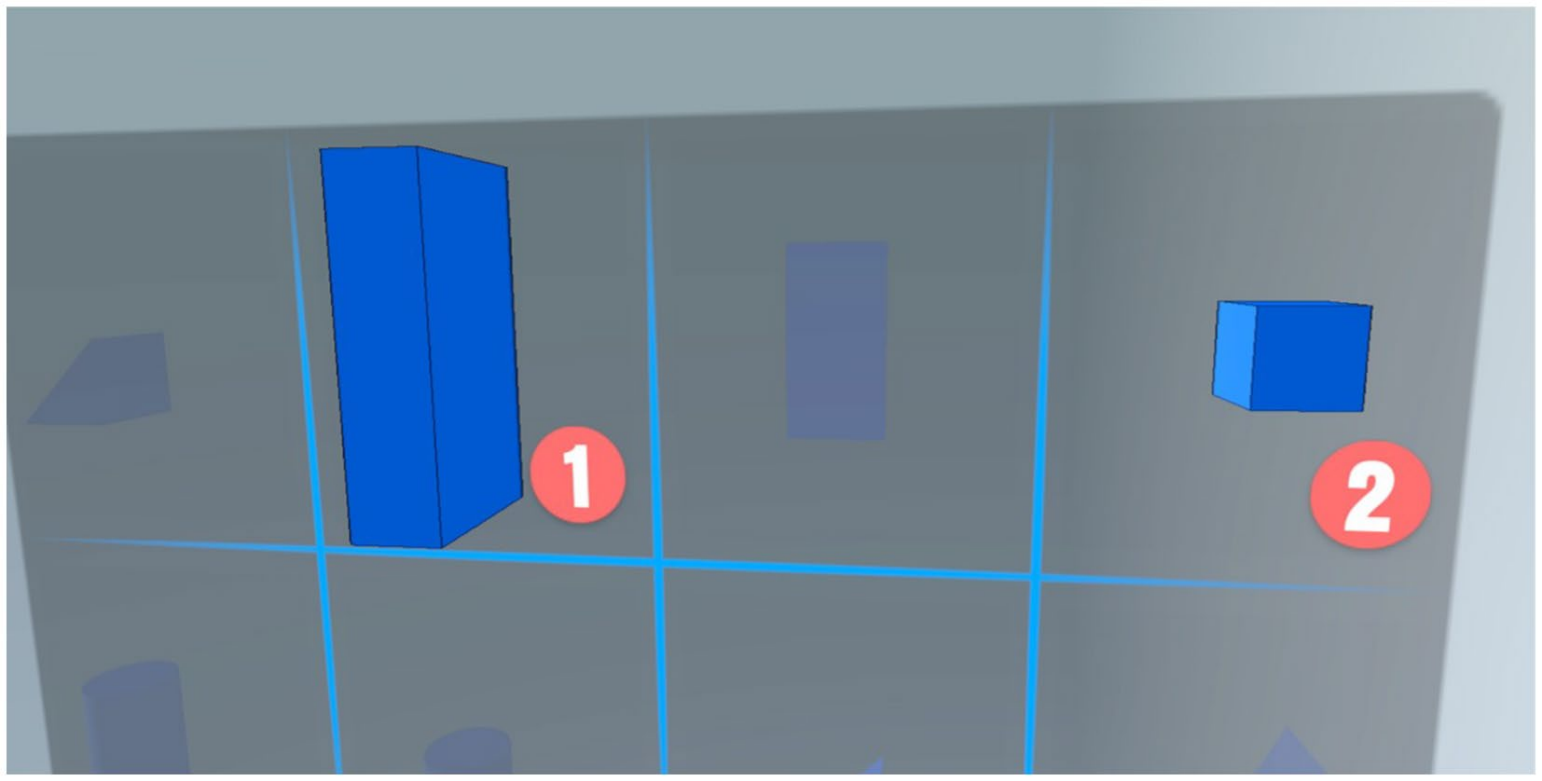
Block option screen of the VR spatial performance assessment system.

The list of blocks in the game stage shows the available blocks and the number of blocks in the stage, while the dark spaces are the blocks that will not be used in the stage, as shown in Figure 7.

**Figure 8:**
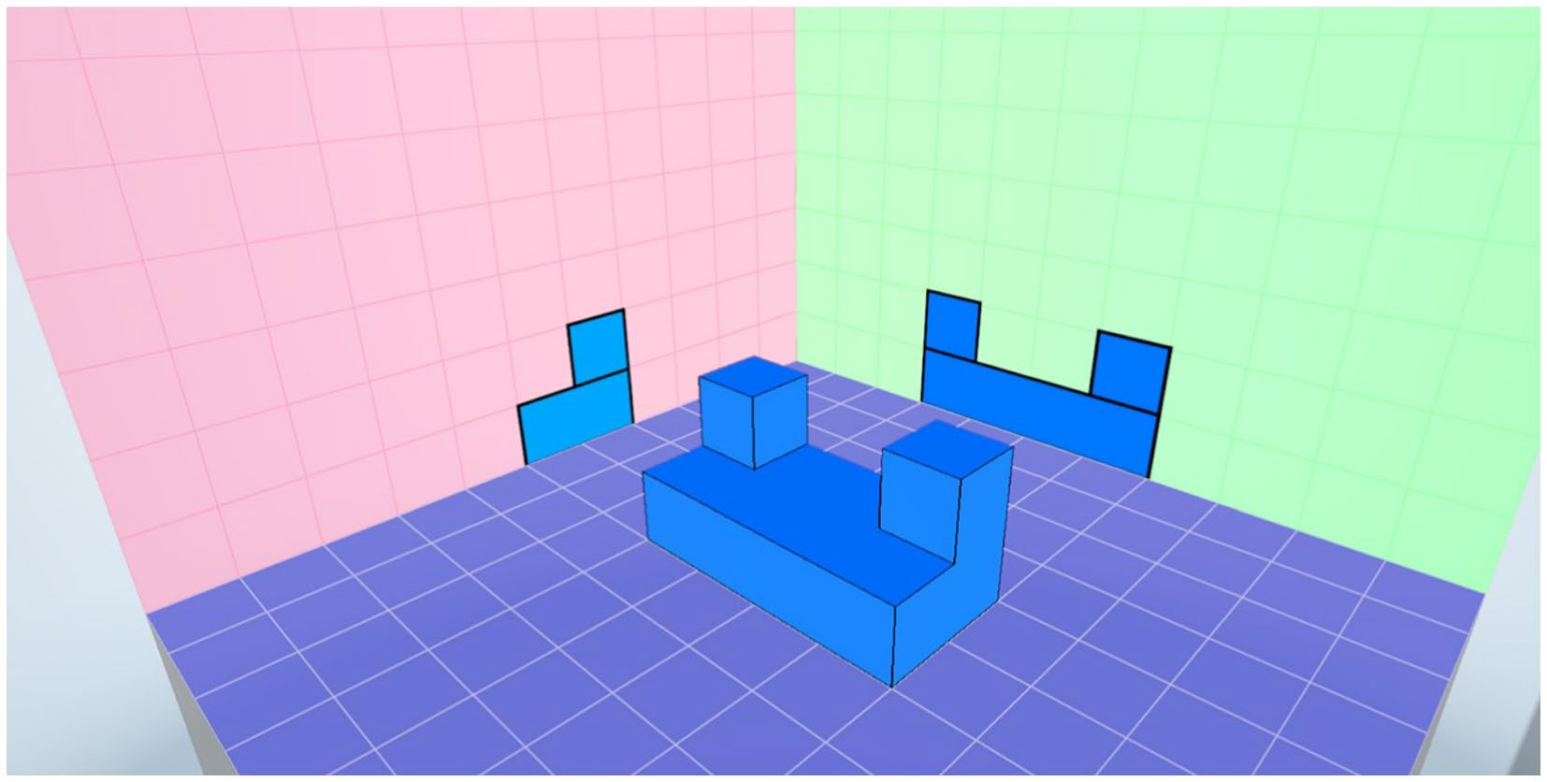
An exemplary screenshot of completing the VR assessment.

Players arrange the schematic diagram of the completed three-dimensional block pattern, and the block positions should correspond exactly to the two views. The placement should not be lower than the platform, as shown in Figure 8.

## Results

### Item Analysis

To ensure the item suitability in each construct, the present study first analyzed the factor loading (FL) of each item; if the FL was lower than 0.5, it was deleted. Second, first-order confirmatory factor analysis (CFA) was applied to test the internal validity of each item by deleting those with the highest residual value in each construct until they met the suggested thresholds (Hair et al., 2019). According to the principle of the independence of the streamlined model and residuals, the *χ*^2^/*df* value should be less than 5, the RMSEA should be less than 0.10, the GFI and AGFI values should be greater than 0.80, as shown in Table 1, and the FL value of the items should be greater than 0.50 (Hair et al., 2019). The results of the deletion in this study were that IBAI was reduced from six to five items, and EBSI was reduced from six to four items, as shown in Table 2.

**Table 1:** First-order CFA.

Level of fit	Threshold value	IBAI	EBSI
*χ* ^2^	–	8.70	0.62
*df*	–	5	2
*χ*^2^/*df*	<5	1.74	0.31
RMSEA	<0.10	0.05	0.00
GFI	>0.80	0.99	0.99
AGFI	>0.80	0.96	0.99

**Table 2:** Factor loadings analysis.

IBAI			EBSI		
Item	Before	After	Item	Before	After
IBAI1	0.56	0.56	EBSI1	0.40	Delete
IBAI2	0.77	0.77	EBSI2	0.92	0.92
IBAI3	0.78	0.79	EBSI3	0.93	0.92
IBAI4	0.78	0.78	EBSI4	0.85	0.85
IBAI5	0.42	Delete	EBSI5	0.42	Delete
IBAI6	0.68	0.68	EBSI6	0.56	0.57

### Reliability and Validity Analysis

#### Reliability

The reliability of each variable was evaluated by reliability analysis with the help of Cronbach’s α (Hair et al., 2019). Emerson (2019) states that Cronbach’s α should usually be 0.70 or higher to meet acceptable thresholds. The Cronbach’s α values for this study ranged from 0.84 to 0.88, as shown in Table 2. For the portfolio reliability section, Hair et al. (2019) suggest that composite reliability (CR) values should exceed the.7 threshold. The CR values for this study ranged from 0.84 to 0.90, as shown in Table 3.

**Table 3:** Reliability and Validity Analysis.

Construct	*M*	*SD*	α	*FL*	CR	AVE	*t*
IBAI	3.98	0.71	0.84	0.55~0.78	0.84	0.52	16.71~19.27
EBSI	2.31	0.98	0.88	0.57~0.93	0.90	0.69	18.75~22.69

#### Convergent Validity

Hair et al. (2019) suggested that the FL value of an item should be higher than 0.50 in order to have convergent validity, so any item with a value lower than this should be deleted. In addition, Fornell-Larcker’s criterion for convergent validity requires that the average variance-extracted values (AVE) should be greater than 0.50 in order for the construct to have convergent validity (Cheung and Wang, 2017). The AVE values in this study ranged from 0.52 to 0.69, as shown in Table 3.

#### Intra-Rater Reliability

Intra-rater reliability analyses are important for research as they help to improve the quality of research by providing information about the amount of misinformation in any diagnosis, score, or rating (Vanbelle, 2016), and correlation coefficient values between 0.50 and 0.60 represent acceptable thresholds (Hodgson et al., 2014). The results show that the intra-rater reliability in this study has acceptable agreement, as shown in Table 4.

**Table 4:** Intra-rater reliability analysis.

Scorer	Scorer 1	Scorer 2	Scorer 3
Scorer 1	1		
Scorer 2	0.66^**^	1	
Scorer 3	0.71^**^	0.68^**^	1

** *p* < 0.001.

#### Performance Analysis

The results of the descriptive analysis showed that the participants’ SPA had a mean (*M*) of 9.82 stages, a standard deviation (*SD*) of 1.94 stages, and a median of 10 stages; the participants’ GCP had *M*=3.43, *SD*=0.71, as shown in Table 5, and a median score of 3.33.

**Table 5:** Performance analysis.

Construct	*M*	*SD*	Med.
SPA	9.82	1.94	10
GCP	3.43	0.71	3.33

### Model Fit Analysis

In this study, AMOS 20 was first applied for the overall fitness analysis and then for the final validation of the study model. Due to the large number of fitted statistics considering different aspects of the fit, it is recommended that researchers report multiple fitted statistics in structural equation modeling studies (Thompson, 2000). The recommended values for the fit indicators are *χ*^2^/*df* less than 5, RMSEA less than 0.1, GFI, AGFI, NFI, NNFI, CFI, IFI, and RFI greater than 0.80 (Abedi et al., 2015), and PNFI and PGFI greater than 0.50 (Hair et al., 2019). The fitted index values for this study were *χ*^2^=116, *df*=43, *χ*^2^/*df*=2.70, RMSEA=0.08, GFI=0.93, AGFI=0.89, NFI=0.92, NNFI=0.94, CFI=0.95, IFI=0.95, RFI=0.90, PNFI=0.72, and PGFI=0.60, all of which meet the criteria recommended by scholars and have good model fit.

### Path Analysis

**Figure 9:**
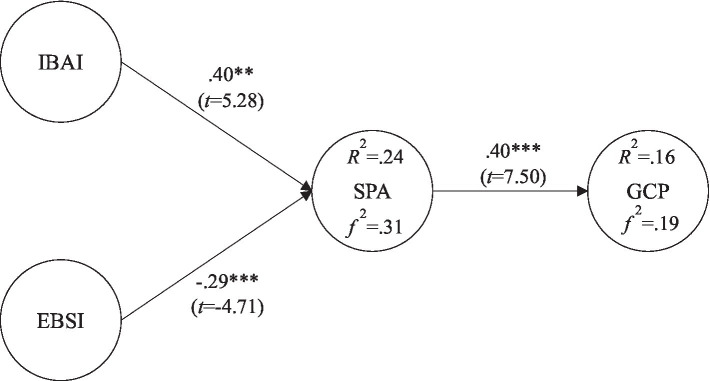
Validation of the research model (^**^p< 0.01; ^***^p< 0.001).

IBAI had a positive effect on SPA (*β*=0.40^**^; *t*=5.28); EBSI had a negative effect on SPA (*β*=−0.29^**^; *t*=−4.71); and SPA had a positive effect on GCP (*β*=0.40^**^; *t*=7.60), as shown in Figure 7. The explanatory power of IBAI and EBSI on SPA was 24% with an *f* ^2^ of 0.31; the explanatory power of SPA on GCP was 16% with an *f* ^2^ of 0.19, as shown in Figure 9.

### Discussion

#### IBAI Can Positively Predict SPA

Research by Kray and Haselhuhn (2007) and Rai and Lin (2019) suggests that learners who hold incremental beliefs of intelligence (IBI) are more engaged and focused on goals because they believe that intelligence is malleable, while research by Costa and Faria (2018) clearly indicates that mastery of incremental intelligence theory has a positive effect on learning success.

In addition, Lüftenegger et al. (2019) also indicated that learners who uphold the belief of intelligence development will want to increase their abilities through continuous efforts because they believe that abilities can be improved through acquired efforts. Additionally, studies by Liu et al. (2014) and Mangels et al. (2006) indicated that if learners tend to support the IBI, this belief will motivate them to face challenges, so they will also tend to set goals that can improve their abilities over time and try to improve their abilities when they encounter difficulties. Also, when they encounter difficulties, they will look for solutions. It is evident that learners who identify with IBI are challenged to perform at their best when faced with tasks, and therefore, learners who hold IBI generally perform better.

Braasch et al. (2014) also indicated that because proponents of IBI view intelligence as malleable, learners who support IBI are committed to improving their intelligence in specific domains, and therefore, beliefs are believed to be effective in predicting learners’ achievement performance. Moreover, Lou et al. (2017) confirmed that IBI (positive beliefs) has a positive effect on learning outcomes. Taken together, it is clear that when participants have positive IBAI, they perform better in SPA. The results of this study also showed that IBAI had a positive effect on SPA.

#### EBSI Can Negatively Predict SPA

According to Dweck et al. (2008), the ITI can influence learners’ learning behaviors positively as well as negatively. Especially, when learners perceive their intelligence to be crystallized, they may fear that the products they use are too complex, and so they lack motivation to learn (Sharifi and Palmeira, 2017). In addition, Bruning et al. (2011), Haimovitz et al. (2011), and Zonnefeld (2019) also suggest that proponents of crystallized intelligence beliefs are not committed to learning to improve their intelligence because they believe that an individual’s intelligence is not malleable, does not increase over time, and does not grow through effort and learning. Therefore, Dweck and Leggett (1988) also showed that supporters of the belief in the solidification of intelligence have lower performance when faced with failure.

Besides, Mangels et al. (2006) showed that people who believe that intelligence is fixed tend to emphasize “performance goals,” which makes them vulnerable to negative feedback, and they possibly avoid challenging learning opportunities as a result. Therefore, Lou et al. (2017) suggested that entity belief of intelligence is often associated with negative learning experiences or performance. From the above, it is possible that proponents of intelligence-consolidation beliefs, when confronted with challenging tasks, may believe that their intelligence or abilities cannot be expanded and do not actively challenge themselves Therefore, when participants hold higher levels of perceptions of EBSI, their cognitive performance in terms of spatial ability may also be poor. The results of this study showed that EBSI had a negative effect on SPA.

#### SPA Can Positively Predict GCP

Stones and Cassidy (2010) and Yokochi and Okada (2005) point out that design is seen as a process in which designers construct their own work, which involves reflecting on the design situation, and before completing the work, designers gradually form mental images before drawing the complete design. Therefore, graphic design is regarded as a complex two-way cognitive process. It is clear that good cognitive ability is essential for design creation. The studies of Dorst and Cross (2001) and Lang et al. (2002) confirm that creative design involves continuous exploration of problems and development of solutions over a period of time, so the design activity is considered to be an activity involving needs analysis and problem clarification, information collection and research, conceptualization and creative thinking, information generation and analysis, evaluation, and optimization of the activity process. Thus, the creative design process involves a large number of cognitive processes, so when the designer has a good cognitive mechanism or cognitive ability, he or she can have a better creative expression of the graphics. In addition, spatial ability is considered as a cognitive ability (Tarampi et al., 2016), and good cognitive ability will have a significant impact on the creative process. As Kell et al. (2013) showed, spatial ability plays a critical and unique role in constructing many important psychological phenomena, and spatial ability also has a unique role in the development of creativity. Design creation can be seen as a creative process, so when participants have high levels of spatial ability, they will have better creative expressions of graphics. The results of this study show that SPA has a positive effect on GCP.

## Conclusions and Recommendations

### Limitations and Further Study

According to cognitive load theory, when visuals (e.g., pictures, diagrams, and animations) are used in conjunction with synchronized text, they can distract students from their visual attention during the learning process (Park et al., 2015). It has also been shown that pictures may place a greater external burden on vision (Crooks et al., 2012), so instructional materials should be designed to avoid unnecessary demands on working memory (Eitel et al., 2014).

Cognitive thinking styles are considered as important attributes influencing an individual’s creativity, meaning that analytical or holistic thinking styles influence the development of creativity (Nisbett et al., 2001). However, there are limited studies on the relationship between various cognitive styles and creativity, and contradictory results have been found (Pitta-Pantazi et al., 2013). Therefore, subsequent research may investigate the effects of cognitive style on spatial ability and attentional performance as well as graphic creativity performance.

### Implications

In the past, psychological or cognitive tests were often used to assess people’s cognitive performance using paper-based tests, and these tests supported the reliability and validity of the measuring instruments. However, with the advancement of technology, immersive cognitive tests based on emerging technologies are becoming more popular and highly discussed. Therefore, research on the application of emerging technologies to cognitive tests will help to expand the understanding of diverse and technological cognitive tests. The results of this study contribute to the practical application of emerging technologies and cognitive measures. Moreover, with the affordability, portability, and quality of VR technology, it has been effectively integrated into educational settings and can meet the outcomes of rigorous assessment of cognitive training (Coleman et al., 2019). Therefore, it is recommended that technical high schools can increase the use of VR. That is, VR system spatial assessment can be used as a tool to assess learners’ spatial performance and can also be used as a learning tool for students in formal courses.

Dweck’s ITI, which explain people’s views on intelligence and how they manifest their behavioral beliefs, have been widely applied in the field of education but less frequently mentioned in the field of design. In this study, the positive and negative effects of beliefs about the development of intelligence and beliefs about the solidification of intelligence on learners’ cognitive performance were found to be helpful for teachers’ development of learners’ cognitive abilities. Thus, a practical implication is that design teachers can assess students’ intellectual beliefs in the early stages of teaching and actively promote students’ IBAI; on the other hand, students’ EBSI can be changed from higher level to lower level by having more practice on various spatiality devices.

### Conclusion

From a cognitive perspective, executive functioning encompasses high-level goal processes that both control goal-directed behavior and respond adaptively to novel, complex, or ambiguous situations. Executive functioning is defined as a set of basic cognitive abilities for goal-directed behavior and has been used as a strong predictor of academic success. Therefore, in order for people to demonstrate good creativity, they need to have high levels of cognitive ability and executive functions, such as thinking. Thus, this study proposes a research model and three research hypotheses based on the concept of executive function and ITI. The study investigated the relationship between students’ IBAI, EBSI, SPA, and GCP. The results of the study showed that: (1) IBAI is positively related to SPA; (2) EBSI is negatively related to SPA; and (3) SPA is positively related to GCP. Additionally, in design courses, one of the most important criteria for performance quality seems to be the creativity of the product. However, from the results of the study, the GCP scores of the participants were in the middle of the range, indicating that there is still much room for growth. However, fortunately, the perceived level of IBAI in this group of young students was higher, and the perceived level of EBSI was also lower.

## Data Availability Statement

The original contributions presented in the study are included in the article/supplementary material, further inquiries can be directed to the corresponding author.

## Ethics Statement

The study was approved by the Ethical Committee of National Taiwan Normal University and students were aware that they were taking part in an evaluation study and that the data they provided was anonymous. Moreover, they could withdraw to answer questions, if they felt uncomfortable. Written informed consent was obtained from each participant before participation in the study.

## Author Contributions

J-CH and J-HY: concept and design. J-HY, J-NY, and L-WK: acquisition of data. J-HY, M-LC, and J-NY: drafting of the manuscript. J-CH and J-HY: critical revision of the manuscript. J-HY and L-WK: statistical analysis. All authors contributed to the article and approved the submitted version.

## Conflict of Interest

The authors declare that the research was conducted in the absence of any commercial or financial relationships that could be construed as a potential conflict of interest.

## Publisher’s Note

All claims expressed in this article are solely those of the authors and do not necessarily represent those of their affiliated organizations, or those of the publisher, the editors and the reviewers. Any product that may be evaluated in this article, or claim that may be made by its manufacturer, is not guaranteed or endorsed by the publisher.

## Footnotes

^1^Ferg, T. (2019). *Creativity advances logical, spatial, and aesthetics cognitive abilities through self-reflection and may improve spatial sequencing memory* (Unpublished master’s thesis). University of Denver, CO
